# Gene expression profiling of primary cultures of ovarian epithelial cells identifies novel molecular classifiers of ovarian cancer

**DOI:** 10.1038/sj.bjc.6602933

**Published:** 2006-01-17

**Authors:** C Le Page, V Ouellet, J Madore, F Ren, T J Hudson, P N Tonin, D M Provencher, A-M Mes-Masson

**Affiliations:** 1 Centre de Recherche of the Center hospitalier de l’Université de Montréal (CR-CHUM)/Institut du cancer de Montréal, Montreal, Quebec, Canada H2L 4M1; 2 Departement de medicine, Université de Montréal, Montreal, Quebec, Canada H2L 4M1; 3 McGill University and Genome Quebec Innovation Center, Montreal, Quebec, Canada H2L 4M1; 4 Departments of Human Genetics and Medicine, McGill University, Montreal, Quebec, Canada H2L 4M1; 5 The Research Institute of the McGill University Health Centre, Montreal, Quebec, Canada H2L 4M1; 6 Division de gynecologie et obstetrique/Université de Montréal, Montreal, Quebec, Canada H2L 4M1

**Keywords:** microarray, ovarian cancer, molecular marker, mesothelin, BMP-2

## Abstract

In order to elucidate the biological variance between normal ovarian surface epithelial (NOSE) and epithelial ovarian cancer (EOC) cells, and to build a molecular classifier to discover new markers distinguishing these cells, we analysed gene expression patterns of 65 primary cultures of these tissues by oligonucleotide microarray. Unsupervised clustering highlights three subgroups of tumours: low malignant potential tumours, invasive solid tumours and tumour cells derived from ascites. We selected 18 genes with expression profiles that enable the distinction of NOSE from these three groups of EOC with 92% accuracy. Validation using an independent published data set derived from tissues or primary cultures confirmed a high accuracy (87–96%). The distinctive expression pattern of a subset of genes was validated by quantitative reverse transcription–PCR. An ovarian-specific tissue array representing tissues from NOSE and EOC samples of various subtypes and grades was used to further assess the protein expression patterns of two differentially expressed genes (Msln and BMP-2) by immunohistochemistry. This study highlights the relevance of using primary cultures of epithelial ovarian cells as a model system for gene profiling studies and demonstrates that the statistical analysis of gene expression profiling is a useful approach for selecting novel molecular tumour markers.

Epithelial ovarian cancer (EOC), a complex disease, is the second most common gynaecological cancer, and accounts for nearly half of the deaths associated with gynaecological pelvic malignancies. Largely asymptomatic, over 70% of patients with ovarian cancer are already at an advanced stage of the disease at initial diagnosis. Considering the morphological, anatomical and clinical differences between tumours, three main classification parameters have emerged, which are based on histological subtype (serous, endometrioid, clear cells, mucinous, Brenner), disease stage (I–IV) and tumour grade (0–3). However, early detection of ovarian cancer is rare, and screening program in the general population have been unsuccessful. Recent studies have focused on gene expression patterns observed for multiple tumours to identify the molecular events involved in the development of cancer in order to uncover diagnostic and prognostic markers as well as new therapeutic targets. Promising results have been reported in several cancers, including bladder, colon and breast cancers (Neibergs *et al*, 2002; van’t Veer *et al*, 2002; Dyrskjot *et al*, 2003), of gene expression profiling as a new means for identifying diagnostic and prognostic targets. While this approach has been applied to ovarian cancer (Adib *et al*, 2004; Donninger *et al*, 2004; Hibbs *et al*, 2004; Lancaster *et al*, 2004; Lee *et al*, 2004; Lu *et al*, 2004; Santin *et al*, 2004; Warrenfeltz *et al*, 2004; Zhang *et al*, 2005), resulting in the identification of several hundred genes differentially expressed between NOSE (normal ovarian surface epithelia) and EOC, less than 10% of these genes were identified in common by at least two reports (Le Page *et al*, 2004). Although sample size, methods of evaluation and expression platforms vary and could account for differences in gene expression profiles, additional independent studies are required for cross-comparison to identify reliable gene markers that vary in expression.

Gene profiling by microarray studies can also be applied to tumour classification, identification of tumour subtypes, or used to predict disease outcome. Expression-based classification schemes have been applied to distinguish ovarian cancers from other tumour types (Golub *et al*, 1999; Ross *et al*, 2000; Ramaswamy *et al*, 2001), but rarely to distinguish normal samples from ovarian cancers. A recent study classified pooled normal epithelial samples from ovarian tumours based on the gene expression of CLDN3 and VEGF (Lu *et al*, 2004). The choice of model system to study molecular profiling is a critical factor that may account for variations in gene expression patterns. As normal epithelial cells are generally quiescent, differential gene expression observed between tissues of NOSE and EOC may reflect differences not necessarily related to oncogenic transformation. In ovarian cancer, primary cultures derived from NOSE and tumour tissue have been described (Lounis *et al*, 1994). In the case of EOC tissues, primary cultures have the advantage of being relatively free of non-transformed cell population. NOSE cultures, derived by scraping the surface of the ovary, are highly homogenous and provide conditions in which normal cells replicate over a limited number of generations. In addition, a recent study comparing candidate genes able to stratify low- from high-malignancy tumours (identified in primary cultures and tumour tissues) showed that candidate genes identified in primary cultures were also able to reclassify samples derived from tumour tissues (Ouellet *et al*, 2005). Based on these advantages, we studied primary cultures enriched over several passages. Using 65 independent samples, we identified gene expression patterns by Affymetrix oligonucleotide microarray analyses, which enabled the stratification of NOSE from EOC and was further supported by tissue array analyses.

## MATERIALS AND METHODS

### Patients, cell culture and clinical material

Tissue samples and sera were obtained with informed consent from participants. Tumour samples were collected from surgeries performed at the Centre Hospitalier de l’Université de Montréal (CHUM). Histopathology, grade and stage of tumours were assigned according to the International Federation of Gynecology and Obstetrics (FIGO) criteria. Normal tissues were obtained from tumour-free participants that have undergone oopherectomies. Primary cell cultures from NOSE and EOC samples were established as described (Kruk *et al*, 1990; Lounis *et al*, 1994) and used for microarray analysis. Cells in primary culture were maintained in OSE media consisting of 50 : 50 medium 199:105 (Sigma, St Louise, MO, USA) supplemented with 10% fetal bovine serum, 2.5 *μ*g ml^−1^ amphotericin B and 50 *μ*g ml^−1^ gentamicin (Kruk *et al*, 1990). The samples used for microarray, reverse transcription (RT)–PCR and tissue array immunohistochemistry (IHC) studies are presented in Table 1.

### RNA preparation and microarray

Total RNA was extracted with TRIzol™ reagent (Gibco/BRL, Life Technologies Inc., Grand Island, NY, USA). RNA was extracted directly from cells grown to 80% confluency. RNA quality was monitored by agarose gel electrophoresis, and independently by the 2100 bioanalyzer using the RNA 6000 Nano LabChip kit (Agilent Technologies, Germany). Biotinylated hybridisation target was prepared from total RNA as described (Tamayo *et al*, 1999). Affymetrix HuFL arrays were used to hybridise label targets, and gene expression levels were calculated for each EST from the scanned image by the Affymetrix GeneChip MAS4 software. This gene chip contains 6800 probe sets representing known genes and ESTs (Affymetrix.com). Microarray experiments were performed at the McGill University and Genome Québec Innovation Centre. The detailed protocol is available at www.genomequebec.mcgill.ca/center.php. The raw data of each experiment, including data used for training and test sets, were normalised according to the mean of the global intensity expression values adjusted to 100 units. After normalisation, we considered all values below 20 as technical noise and rescaled these values to 20. Next, we removed for further analysis all expression values for a given probe set with a poor reliability score or ‘A’ call (ambiguous signal) that occurred across all samples. Using this filtering approach, the expression values of 4888 probe sets were examined for patterns of gene expression. Values were then converted into log_10_ for further analysis. Both the raw and normalised data sets are available at http://www.genomequebec.mcgill.ca/ovarian/.

### Clustering

We carried out hierarchical clustering analysis using GeneSpring™ software (Silicone Genetics) on the filtered data set. We used the distance branch of 0.1 with a Pearson correlation as a similarity metric.

### Supervised class comparison

Three statistical tests were used to identify classifiers. The signal-to-noise metric *S*_*x*_=(*μ*_1_−*μ*_2_)/(*σ*_1_+*σ*_2_) was applied, where *μ* represents the mean and *σ* the standard deviation of either class 1 or 2 (Golub *et al*, 1999). Here, class 1 represents NOSE samples and class 2 represents tumour samples. Only genes with ∣*S*_*x*_∣⩾0.05 were selected. Classifiers were also identified using the significance analysis of microarray (SAM) software described (Tusher *et al*, 2001) and available at http://www-stat-class.standford.edu/SAM/SAMServlet. One thousand permutations were applied in an unpaired filtered data set. The threshold was chosen according to a determined false discovery rate (FRD<5%). A non-parametric test was also performed using the Mann–Whitney *U*-test included in the GeneSpring™ software, including an FDR correction (*P*-value <0.05 or *P*<0.15) (Silicone Genetics).

### Tumour classification and prediction

To classify samples, we used a *k*-nearest neighbour algorithm included in the GeneSpring™ software (Silicon Genetics). Leave-one-out cross-validation approach was used to evaluate the predictors in the training set. A class prediction test was used to predict the independent test samples based on classifiers defined from the training set. Each sample from the test set is classified by finding the *k*-nearest neighbouring training samples. The neighbour number was *n*−1, where *n* is the smallest number of samples in one group. The decision cutoff was *P*<0.2. All sample sets (training and test sets) were evaluated with the same algorithm.

### Quantitative PCR

As previously described (Ouellet *et al*, 2005), RNA was linearly amplified by Alethia Biotherapeutics (Montreal, QC, Canada). The RAMP RNA produced is (+) sense. The cDNA synthesis was performed according to the protocol of the SuperScript™ First-Strand Synthesis System for RT–PCR (Invitrogen Life Technologies) with a starting amount of 2 *μ*g of RNA; the RT was performed with random hexamers. The condition of the PCR reaction (temperature, specificity) was defined by conventional PCR. Positive and negative controls were introduced in all experiments. Quantitative PCR (Q-PCR) was performed using Rotor-gene 3000 Real-Time Centrifugal DNA Amplification System (Corbet Tumor Tissues Research, NSW, Australia). We used the Quantitect™ SYBR Green PCR (Qiagen Inc., ON, Canada) reaction mixture according to the manufacturer's instructions. Serial dilutions were performed to generate a standard curve for each gene tested in order to define the efficiency of the Q-PCR reaction, and a melt curve was constructed to confirm the specificity of the reaction. Experiments were performed in duplicate. Control RNA (Erk) was chosen based on stable expression in 69 ovarian samples containing normal and tumour types as previously described (Ouellet *et al*, 2005). Primers are described in Supplementary Table 2. We used the Pfaffl analysis method to measure the relative quantity of gene expression (Pfaffl, 2001). Statistical analyses were performed using the Student's *t*-test.

### Tissue array and IHC

The following monoclonal antibodies were used in IHC: anti-ATPase*β*1, anti-BMP-2, anti-TNFR1 (Santa-Cruz Biotechnology, CA, USA) and anti-mesothelin (Msln) (Biogenex, San Roman, CA, USA). A tissue array containing 94 cores of ovarian epithelial tissues (Table 1) was amassed and used for IHC studies. Briefly, the tissue array was heated at 60°C for 30 min, de-paraffinised in toluene and rehydrated in a gradient of ethanol. To unmask antigen, the slides were submerged in 90°C citrate buffer (0.01 M citric acid+500 *μ*l Tween 20 l^−1^ adjusted to pH 6.0) (JT Baker, Philipsburg, NJ, USA) for 15 min. The tissue was blocked with a protein-blocking serum-free reagent (DakoCytomation Inc., Mississauga, ON, Canada) and incubated with different antibodies overnight at 4°C in a humid chamber. The optimal concentration for each primary antibody was determined by serial dilutions. Subsequently, endogenous peroxidase activity was quenched by treatment with 3% H_2_O_2_. The array was then incubated with a secondary biotinylated antibody (DakoCytomation Inc., Mississauga, ON, Canada) for 10 min, followed by incubation with a streptavidin–peroxidase complex (Dako Diagnostics Canada Inc., Mississauga, ON, Canada) for 10 min at room temperature. Reaction products were developed using diaminobenzidine (brown stain) containing 0.3% H_2_O_2_ as a substrate for peroxidase, and nuclei were counterstained with diluted haematoxylin (blue stain). Epithelial zones were scored according to the intensity of staining (0 for absence, 1 for very weak, 2 for weak, 3 for moderate and 4 for high intensity). Each array was independently analysed in a blinded study by two independent observers. Statistical analyses were performed using the Mann–Whitney *U*-test.

## RESULTS

### Comparative gene profiling of normal epithelial cells (NOSE) and epithelial ovarian tumours (EOC)

To identify markers that may be associated with the progression towards a malignant phenotype, we used oligo-microarrays to analyse gene expression profiles of normal or tumoral primary cultures of ovarian epithelial cells. Primary cultures were derived from specimens selected on the basis of the disease course before treatment of the patient (e.g. no chemotherapy). Primary cultures from 54 malignant tumours and 11 NOSE samples were analysed by microarray. The samples represent as much as possible the diversity of EOC in terms of grade and histopathology (Table 1); Brenner tumours were not represented in our set.

**Figure 1:**
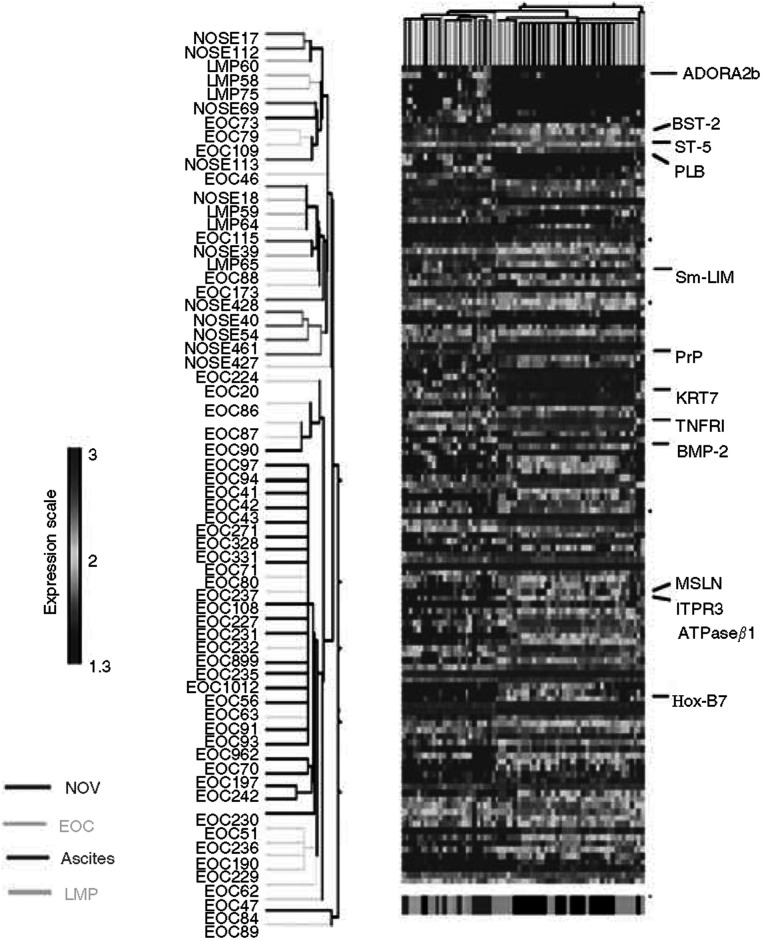
Hierarchical clustering of gene analysis expression of 11 normal ovarian epithelia (NOSE) and 55 epithelial ovarian tumours (EOC) from primary culture. Sample clusters based on 126 genes differentially expressed in tumour samples *vs* normal epithelia. Clustering was carried out based on the genes retrieved by class comparison (*P*-value <0.05, FDR<0.05, ∣*S*_*x*_∣>0.50). Genes were selected by three statistic algorithms (see Materials and Methods). Each row represents a gene and each column represents a sample. Identity of each sample in the clusters is shown on the left of the figure. Colour intensity represents level of gene expression transformed in log 10. LMP=low malignant potential tumours; Msln refers to the protein; MSLN refers to the gene.

To gain insight into genes whose expression is associated with ovarian malignant transformation, we analysed the expression array data derived from HuGeneFL 6800 Affymetrix GeneChip® using three different supervised classification methods. The group of NOSE samples was compared to the 54 EOC samples. A total of 505 genes were selected by at least one algorithm (data not shown), where 126 ESTs were identified in common by all three algorithms that represent genes that were significantly differentially expressed when NOSE samples were compared with EOC samples (Figure 1 and Supplementary Table 1). About 25% of these genes have previously been shown to be differentially expressed in ovarian cancer cells relative to normal ovarian cells (partially shown in Table 2) (Le Page *et al*, 2004). The 126 genes are associated with known biological functions such as angiogenesis, growth and proliferation, signalling, cell adhesion and metastases, and are potential oncogenes and tumour suppressors (Table 2). The expression profile of these 126 genes, subjected to a two-way hierarchical cluster analysis (Figure 1), identified several characteristic profiles that differed among the groups analysed. The NOSE samples clustered together with the low malignant potential (LMP) tumours and three grade 2 tumours, forming a group considered as low-grade samples. The majority of tumours are grouped in a second cluster independently of histopathological type. We also noticed that ascites show a more homogenous profile in comparison to the solid invasive tumours and, clustered in a sub-branch. The unsupervised clustering analysis showed that these 126 genes were not able to discriminate NOSE samples from LMP samples in contrast to invasive EOC samples. This suggests that LMP tumours should be analysed separately from the invasive tumours to identify specific gene markers for low-grade disease.

### Selection of genes distinguishing subclasses of ovarian tumours

**Figure 2:**
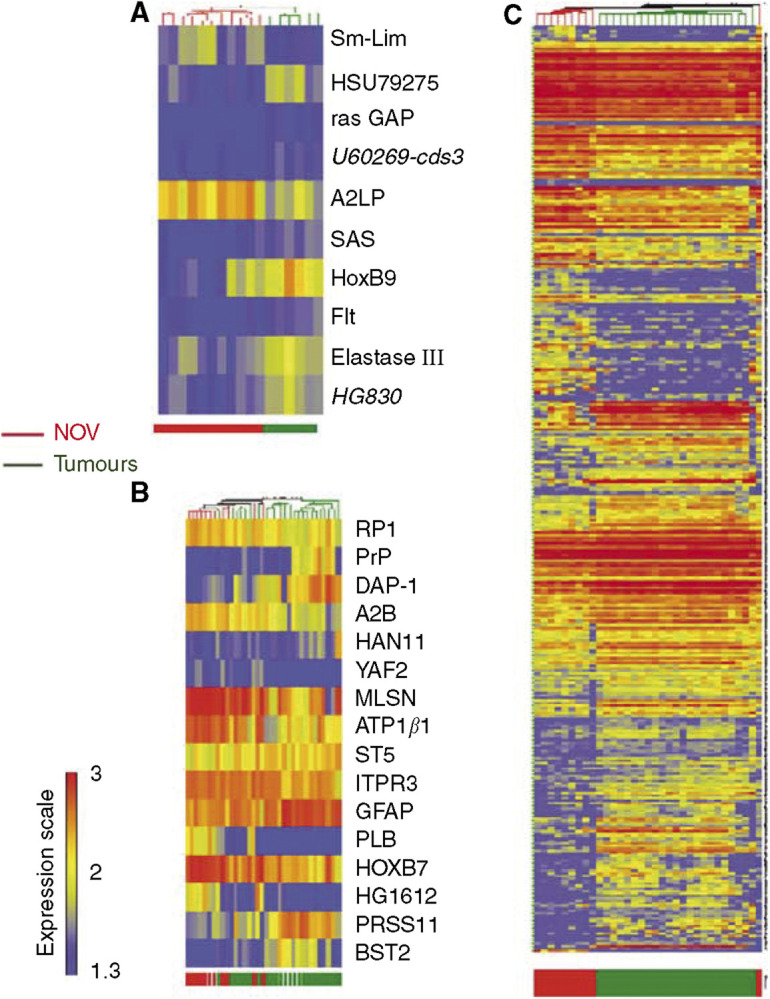
(**A**) Hierarchical clustering of gene analysis expression of 11 normal ovarian epithelia and six EOC of low malignant potential. Sample clusters and gene profile based on 11 genes differentially expressed in LMP samples. Genes were identified using U algorithm (*P*<0.15). (**B**) Hierarchical clustering of gene analysis expression of 11 ovarian epithelia and 24 solid invasive ovarian tumours. Supervised sample clusters and gene profile based on 16 genes differentially expressed in malignant tumour samples are shown. Genes were identified using U algorithm (*P*<0.05). (**C**) Hierarchical clustering of gene analysis expression of 11 ovarian epithelia and 23 ovarian samples from malignant ascites. Supervised sample clusters and gene profile based on 270 genes differentially expressed ascites are shown. Genes were identified using U algorithm (*P*<0.05). Each row represents a gene and each column represents a sample. Colour intensity represents level of gene expression transformed in log 10 corresponding to the expression scale shown on the left side of the figure. NOSE=normal ovarian epithelia; EOC=epithelial ovarian cancer. Colour bar at the bottom of the gene expression matrice shows the class of sample.

Based on the results of our initial analysis, we subsequently used a supervised approach to select genes that were able to differentiate the major groups of tumours (LMP, ascites and solid invasive tumours) that clustered in the unsupervised analysis. For this purpose, the three groups of tumours were individually compared to the NOSE samples. Ten genes were differentially expressed in LMP tumour cells (Figure 2A), 16 genes were differentiated in solid invasive tumours (Figure 2B) and 270 genes were differentially expressed in ascites tumours (Figure 2C). Among these 270 genes were the 16 differentially expressed genes identified in the solid invasive tumour group of invasive tumours.

As there are no common genes found in the independent analyses of LMP, ascites and invasive tumour groups that distinguished them from NOSE samples, we tested as a classified model the 10 genes differentially expressed in LMP combined with the 16 differentially expressed genes found in common in the analyses of ascites and invasive solid tumours. As an objective prediction of low- and high-malignant ovarian tumours using a limited set of genes could be appropriate for clinical use, we selected gene classifiers showing at least a 1.5-fold gene expression difference between NOSE samples and tumour samples. Using this approach, we reduced the original set of 26 markers to a subset of 18 markers that were the most predictive (Table 3). Each of the 65 tumours was reclassified according to the expression pattern of the 18 gene classifiers. This classifier predicted correctly 60 out of 65 samples (92%) (Table 4). All normal and ascites samples were correctly classified, and only three solid tumours (1.5%) were incorrectly classified as NOSE. This classifier showed greater accuracy in comparison to the large set of 126 genes selected in Figure 1B (Table 4). The incorrectly classified samples included one grade B (LMP) and one grade 2 serous tumours as well as one unknown grade tumour. Interestingly, this classifier allowed a lower ratio of errors/correctly classified (*r*=0.05) than the set of 126 genes. To validate the classifier, an additional independent test set of 23 primary culture samples and 137 arrays of surgical specimens from publicly available data sets derived from Affymetrix HuFL GeneChip platform (Welsh *et al*, 2001; Schwartz *et al*, 2002; Ouellet *et al*, 2005) were tested. The set of 23 primary culture samples contained two LMP serous ascites. All these samples were normalised the same way as our training set. The algorithm used for class prediction was the same as previously used (*k*-nearest neighbour). Instead of a cross-validation scheme, these new samples were predicted using a class prediction scheme. The tumour potential was predicted, and resulted in 87% accuracy for the primary culture sample set and 96% accuracy for the tissue sample set (Table 4).

### Validation by Q-PCR

**Figure 3:**
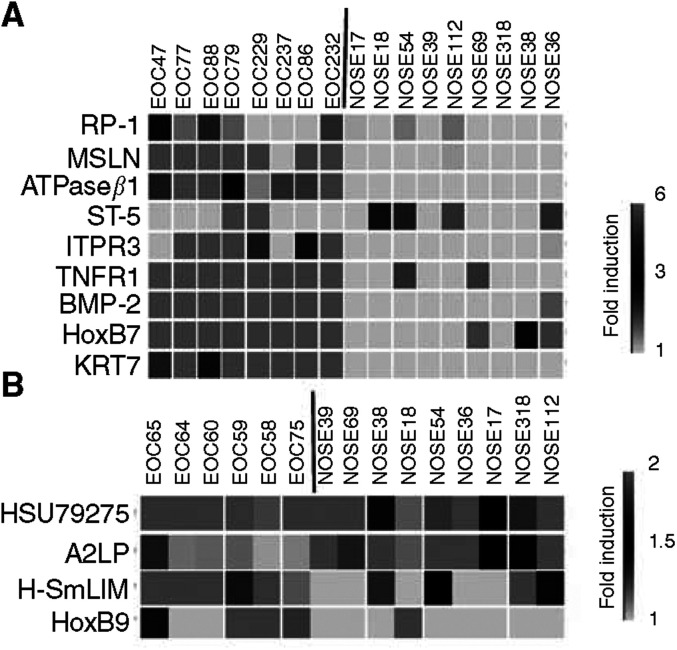
Validation by real-time Q-PCR. A 2 *μ*g portion of RNA extracted from primary culture was retro-transcribed and used for real-time Q-PCR using specific primers for RP-1, MSLN, ATPase*β*1, HoxB7, ST-5, ITPR3, TNFR1, KRT7 and BMP-2 in eight malignant samples and nine NOSE (normal ovarian surface epithelia) (**A**), A2RP, HSU79271, HoxB9 and SmLIM in six LMP (low malignant potential) samples and nine NOSE (**B**). Each expression level was normalised to that of the control RNA. Relative fold change expression is the ratio of the 61 NOSE gene expression to that of other samples. Owing to the downregulated profile of ST-5 gene expression, PCR was performed using EOC908 as reference. Green colour represents expression ratio lower than 1, black represents expression ratio equal to 1 and red represents expression ratio higher than 1.

To validate the differential expression observed by DNA microarray, we tested the expression of 13 markers encoding known proteins by real-time Q-PCR. This set of 13 markers was composed of a subset of 10 markers selected from the classifier analysis (MSLN, RP-1, ITPR3, HoxB7, ATPase*β*1, ST-5, HoxB9, SmLIM, A2LP and HSU79271) in addition to three genes (TNFRI, KRT7 and BMP-2) that not only differentiate NOSEs and EOCs but also reveal differences between ascites and solid tumours (Figure 1 and Supplementary data 1). RNA from primary cultures of nine NOSE, eight malignant EOC or six LMP tumours already used for the profiling analysis was randomly chosen as templates for these assays. As shown in Figure 3, in general, Q- PCR results were consistent with expression microarray data patterns. A significant difference (*P*<0.05) was noticed between the expression levels of NOSE and EOC for each of the tested genes, with the exception of RP-1 and SmLIM (Figure 3A and B, respectively).

We chose the 11 genes that were validated by Q-PCR as differentially expressed between NOSE and EOC, and tested whether their relative RNA expression monitored by Q-PCR could classify the samples using the ‘leave-one-out’ cross-validation scheme. All tumours were correctly classified; however, two NOSE samples were indistinguishable from LMP, giving a total of 87% accuracy. Even with a small set of samples, these results showed that PCR classification using a smaller set of marker genes could reach the accuracy rate obtained by microarray data.

### Immunochemical expression of ATPase*β*1, TNFR1, BMP-2 and Msln in ovarian tumours

**Figure 4:**
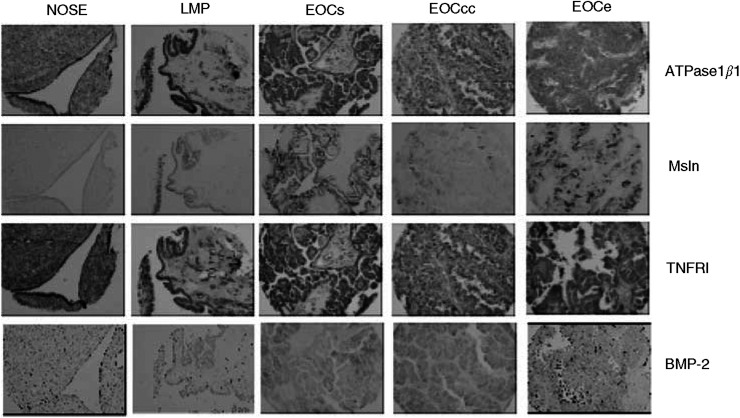
Immunohistochemistry of NOSE and EOC tissues. Expression of ATPase*β*1, MSLN, TNFR1 and BMP-2 in normal ovarian surface epithelium (NOSE), low-malignant (LMP) and high-malignant serous (EOCs), clear cell (EOCcc) and endometrioid (EOCe) ovarian cancer tissues. Keratin 19 is strongly expressed in all three tissue types. ATPase*β*1 and TNFRI are expressed in all tissue types. Mesothelin and BMP-2 are weakly expressed in NOSE tissues, whereas they are more strongly expressed in EOC tissues. Note the apical expression of Msln.

We also determined whether the protein encoded by the classifier genes had similar classifying potential. For this purpose, we used immunohistochemical assays on a tissue array containing 20 NOSE and 69 EOC cores (Table 1). This assay was restricted by the commercial availability of antibodies and thus we were not able to test any of the seven of the 10 LMP classifying genes. Among the 11 classifiers of highly malignant tumours, we were able to test ATPase*β*1 and Msln expression. We also immunostained the tissue array with keratin 19 as a positive control to visualise epithelial cells. ATPase*β*1 was expressed at a high level in both NOSE and EOC (Figure 4). Although IHC detected apparently high levels of expression of ATPase in both tissues, it was unable to discriminate the samples based on the intensity of staining. In contrast to ATPase*β*1, Msln protein was weakly expressed in some normal epithelial cells (Table 5) and more strongly in cancerous cells (Figure 4). As already described (Chang *et al*, 2003; Ordonez, 2003; Drapkin *et al*, 2004), Msln staining was observed on the apical layer of tumour tissues (Figure 4). The intensity of staining was correlated with the highest-grade tumours, where a significant difference was observed between normal epithelia and grade 2 or grade 3 tumours (*P*<0.03; Table 5). However, all serous tumours stained for Msln regardless of the malignancy (Table 5), supporting the idea that Msln is a marker of serous tumours independently of the grade and stage.

We also tested the expression of TNFR1 and BMP-2, two upregulated genes in tumour ascites cells relative to NOSE samples. As observed with ATPase*β*1, TNFR1 was expressed at a high level in both NOSE and EOC samples (Figure 4) and was unable to discriminate the samples based on the intensity of staining. In contrast, when expressed in NOSE tissues, the expression of BMP-2 was globally weaker than in tumour tissues (Table 5 and Figure 4). In contrast to Msln expression, no correlation between tumour grade and BMP-2 expression was observed, and the clear cell tumours showed a stronger staining than endometrioid and serous tissues. We also estimated the performance of BMP-2 as an individual marker of ovarian cancer. Threshold of moderate staining (3+) yielded the best possible sensitivity and specificity values to predict the tumoral status potential.

## DISCUSSION

Owing to the complexity of ovarian cancer and, in particular, the presence of mixed subtypes, there is considerable interest in defining a molecular signature for ovarian cancer. Using microarray analysis, we were able to identify distinctive profiles of gene expression of ovarian cancer cells. These profiles distinguished LMP tumours, malignant tumours and malignant cells from ovarian ascites. These three groups of ovarian cancer cells are currently distinguishable by their invasive potential but not by histopathologic subtypes as previously observed (Schaner *et al*, 2003). Low malignant potential tumours are the least aggressive tumours of the studied groups and rarely invade the peritoneal cavity or migrate to the omentum. In contrast to solid malignant tumours that invade the stroma of the ovary and may be associated with local sites of progression, ascites tumour cells may implant at distal sites in the peritoneal cavity. The ability, in our study, to distinguish solid tumours *vs* ascites based on gene expression signatures may reflect different biological characteristics of these cells associated with the invasiveness and migration potential, as highlighted by the number of genes associated with angiogenesis, adhesion and metastasis processes (Table 2).

We also evaluated the classification ability of the expression of several genes detected by microarray. The predictive property of the gene set classifier may have considerable clinical importance if validated in a large set of tissue samples. Indeed, we were able to correctly classify approximately 90% of the samples. This represents a result comparable to what has been obtained with other types of cancer (Golub *et al*, 1999; Beer *et al*, 2002; Shipp *et al*, 2002; van’t Veer *et al*, 2002; Dyrskjot *et al*, 2003; Gordon *et al*, 2003; Simon, 2003), and represents the first report of this type in ovarian cancer. The success of our approach may in part be explained by the choice of the model system, which does not rely on primary undissected tumour tissue that may contain several cell types. In particular, we have previously demonstrated that even short-term passage of primary cultures results in an enriched homogeneous cell population (Lounis *et al*, 1994). The absence of non-malignant contaminating cells in our samples has probably allowed a strict selection of genes specifically expressed in epithelial cells. The second reason is the strategy of using the combination of small sets of specific genes to build a classifier able to differentiate simultaneously low- and high-grade malignant tumours. Gene-level expressions observed by Q-PCR confirmed the usefulness of this method and allowed for the distinction of tumour samples, which not only validated the biological relevance of the gene markers but also supported the use of Q-PCR as a new diagnostic/prognostic tool for determining tumour class. Although tumour classification based on gene expression detected by RT–PCR has already been successfully applied in mesothelia cancer (Gordon *et al*, 2003), the validity of this method for ovarian cancer has to be confirmed prospectively in a larger set of patient specimens.

Interestingly, our LMP tumours showed the least distinct profile in comparison to NOSE (Figure 1), which correlates with the weak aggressive potential of these tumours and the favourable prognostic for the patient. The present results correlate well with recent attempts to distinguish LMP and solid malignant tumours using a microarray and gene profiling approach (Gilks *et al*, 2005; Meinhold-Heerlein *et al*, 2005; Ouellet *et al*, 2005). Here, we detected only a very small set of genes differentially expressed in LMP tumours. Those genes are genes encoding for unknown proteins such as HSU79275, U60269, HG830-HT830, and genes encoding for proteins without a clear function related to oncogenesis, with the exception of the gene SAS (sarcoma amplified sequence). Owing to its close localisation with MDM2, an inhibitor of p53, the SAS oncogene is often associated with the amplification of MDM2 in human sarcomas (Meltzer *et al*, 1991). In ovarian cancer expression, MDM2 is more characteristic of serous LMP (Palazzo *et al*, 2000). A deeper analysis would be necessary to determine the potential involvement of SAS and MDM2 and their prognostic value in serous LMP cancer. In addition, the small number of genes detected as differentially expressed between LMP and NOSE suggests that these tumours have a very similar profile to NOSE cells, and also suggests that a better molecular distinction between NOSE and LMP will need further investigation.

In the context of tumour suppressors involved in ovarian cancer, much attention has focused on the role of BRCA and p53. Whereas p53 mutation appears to be frequent (Marks *et al*, 1991; Okamoto *et al*, 1991), BRCA mutation occurs in less than 10% in all diagnosed cases (Ford *et al*, 1994; Cass *et al*, 2003) and represents a small minority of ovarian cancers. Here, we identified ST-5/HTS1 as a tumour suppressor gene downregulated in the majority of ovarian primary cultures derived from malignant tumours. ST-5 has been initially identified as a HeLa tumour suppression gene (Lichy *et al*, 1992; Amid *et al*, 2001). Interestingly, HeLa cells are derived from an uterine tumour, which suggests that ST-5 may be hormonally regulated. However, little is known about the regulation and the expression of this tumour suppressor. Future interests will allow a better understanding of the role of ST-5 in the gynaecological female tract cancers such as ovarian and uterine cancers.

Among the genes differentially expressed in malignant EOC is Msln. Mesothelin mRNA has previously been described as overexpressed in ovarian and mesothelioma cancer tissues, and the gene product is referred to a marker of these two cancers (Ordonez, 2003; Drapkin *et al*, 2004; McIntosh *et al*, 2004). However, these studies analysed the Msln protein expression using a small number of samples, and usually limited their analysis to serous ovarian cancer samples. We extended these observations on a larger set of samples also containing endometrioid and clear cell tissues with different tumour grades. In contrast to the general statement that Msln is a specific ovarian cancer marker, here we observed that normal epithelial cells can at times express Msln and a weak or absent expression is observed in both low-grade endometrioid and clear cell tumours. Our extended analysis would thus emphasise that no unique protein marker may be appropriate to classify such a heterogeneous disease, and reinforces the notion that a combination of protein markers is possibly necessary to allow the correct distinction of normal and cancerous ovarian epithelial cells. Our results support this idea but do not rule out the idea that Msln may be an appropriate ovarian cancer marker in association with other markers, such as BMP-2, that complement staining in low-grade endometrioid and clear cell tumours.

In conclusion, our results indicate that a molecular classification system, based on the statistical analysis of gene expression profiling, is a useful approach for tumour subgrouping and the discovery of new molecular markers. These observations articulate a new area of research in the understanding of ovarian cancer as well as illuminating new therapeutic strategies. Combination of oligo-microarray, RT–PCR and tissue array linked to clinical and pathology data will facilitate rapid characterisation of candidate markers.

## Figures and Tables

**Table 1:** Specimen and clinical data of ovarian cancer patients used in each experiments

		**Grade**					**Stage**			
**Experiments**	**Samples**	**LMP**	**1**	**2**	**3**	**Unknown**	**Low (1–2)**	**High (3–4)**	**Solid tumours**	**Ascites**
Training set (*n*=65)	Normal *n*=11									
	Serous *n*=40	6	1	8	25		4	36	23	17
	Endometrioid *n*=9			3	6		3	6	5	5
	Clear cell *n*=2				2		1	1	0	2
	Mixed *n*=1					1		1	1	0
	Mucinous=1	1					1			
	Undifferentiated *n*=1				3				0	1
	Total tumours *n*=54	7	1	11	37	1	10	44	29	25
										
Test set primary culture (*n*=23)	Normal and benign *n*=6									
	Serous *n*=11	3		3	5		3	7	1	9
	Endometrioid *n*=1				1			1	1	0
	Clear cell *n*=1				1			1	0	1
	Mixed *n*=3				3			3	1	2
	Undifferentiated *n*=1				1			1	1	0
	Total tumours *n*=17	3		3	11		3	13	4	12
Test set tissue (*n*=137)^a^	Normal and benign *n*=5						NS	NS	5	0
	Serous *n*=81	6	3	21	41	10	NS	NS	81	0
	Endometrioid *n*=33		10	10	13		NS	NS	33	0
	Clear cell *n*=8				8		NS	NS	8	0
	Mucinous *n*=10		7	3			NS	NS	10	0
	Total tumours *n*=132	6	20	34	62	10	NS	NS	137	0
Tissue array (*n*=89)	Normal *n*=20									
	Serous *n*=21	4	5	5	7	1	8	13	21	0
	Endometrioid *n*=27		13	7	5	2	17	10	27	0
	Clear cell n=17			5	9	3	11	6	17	0
	Mixed *n*=4				3	1	2	2	4	0
	Total tumours *n*=69	4	18	17	24	6	38	31	69	0

LMP=low malignant potential.

a From publicly available data sets (Welsh *et al*, 2001; Schwartz *et al*, 2002; Ouellet *et al*, 2005). ns: nonspecified.

**Table 2:** Biological function of genes deregulated in EOC

	**Gene ID**	**Unigene_ID**	**Title**	**Gene**	**Regulation in EOC**	***P* a**	**Reported in other studies in relation to ovarian cancer**
Cell cycle/growth	K01911_at	Hs.1832	Neuropeptide Y	NPY	Up	0.0035	No
	D21878_at	Hs.169998	Bone marrow stromal cell antigen 1	BST1	Up	2E−05	No
	U72066_at	Hs.29287	Retinoblastoma-binding protein 8	RBBP8	Up	2E−05	No
							
Oncogenes or tumour suppressors	X77548_at	Hs.99908	Nuclear receptor coactivator 4	NCOA4	Up	0.0005	No
	J04102_at	Hs.85146	v-ets avian erythroblastosis virus E26 oncogene homolog 2	ETS2	Up	0.001	Ni
	L20861_at	Hs.152213	Wingless-type MMTV integration site family, member 5A	WNT5A	Up	3E−05	Yes
	X16662	Hs.87268	Annexin A8	ANXA8	Up	5E−06	No
	U61262_at	Hs.90408	Neogenin (chicken) homolog 1, netrin receptor	NEO1	Up	2E−06	Yes
	U15131_at	Hs.79265	Suppression of tumorigenicity 5	ST5	Down	2E−05	No
							
Angiogenesis	M34539	Hs.752	FK506-binding protein 1A (12kD)	FKBP1A/FKPB12	Up	7E−05	No
	M30257	Hs.109225	Vascular cell adhesion molecule 1	VCAM1	Up	5E−05	Yes
	D49950_at	Hs.83077	Interleukin 18 (interferon-gamma-inducing factor)	IL18	Up	2E−05	Yes
	M31551_s_at	M31551	All_M31551 576-1134, Human urokinase inhibitor (PAI-2) gene	PAI-2	Up	0.0002	Yes
	J04513_at	J04513	Human basic fibroblast growth factor (bFGF/FGF2)	bFGF	Up	0.0002	Yes
	X72012_at	Hs.76753	Endoglin (Osler–Rendu–Weber syndrome 1)	ENG	Down	0.0002	Yes
	M22960_at	Hs.73853	Bone morphogenetic protein 2	BMP-2	Up		Yes
							
Adhesion	D13666		Osteoblast specific factor 2 (fasciclin I-like)	OSF-2	Up	0.0002	Yes
	D84424	Hs.57697	Hyaluronan synthase 1	HAS1	Up	0.0006	No
	M28882	Hs.211579	Melanoma adhesion molecule	MCAM	Down	2.7E−6	No
	M29277	Hs.211579	Melanoma adhesion molecule	MCAM	Down	8.54E−6	No}
	M59911	Hs.265829	Integrin, alpha 3 (antigen CD49C, alpha 3 subunit of VLA-3 receptor)	ITGA3	Up	5E−06	No
	U41767	Hs.92208	A disintegrin and metalloproteinase domain 15 (metargidin)	ADAM15	Up	0.0042	Yes
	Y00097_s_at	Hs.118796	Annexin A6	ANXA6	Down	1E−05	No
	Z26653	Hs.75279	Laminin, alpha 2 (merosin, congenital muscular dystrophy)	LAMA2	Down	4E−05	Yes
	D49950	Hs.83077	Interleukin 18 (interferon-gamma-inducing factor)	IL18	Up	2E−05	Yes
	M30257_s_at	Hs.109225	Vascular cell adhesion molecule 1	VCAM1	Up	5E−05	Yes
	U40282_at	Hs.6196	Integrin-linked kinase	ILK	Down	1E−05	Yes
							
Metastasis	D21337_at	Hs.408	Collagen, type IV, alpha 6	COL4A6	Up	0.001	Yes
	M90657_at	Hs.3337	Transmembrane 4 superfamily member 1	TM4SF1	Up	0.0009	No
	M22489_at	Hs.73853	Bone morphogenetic protein 2	BMP2	Up	0.0006	Yes
	L20861	Hs.152213	Wingless-type MMTV integration site family, member 5A	WNT5A	Up	3E−05	Yes
							
Transduction signal/transcription factor	D25538	Hs.172199	Adenylate cyclase 7	ADCY7	Down	2E−05	No
	L07597	Hs.149957	Ribosomal protein S6 kinase, 90kD, polypeptide 1	RPS6KA1	Up	3E−05	No
	M64497	Hs.288869	Nuclear receptor subfamily 2, group F, member 2	NR2F2	Down	0.0004	No
	U24576	Hs.3844	LIM domain only 4	LMO4	Up	1E−06	No
	U28833	Hs.86724	Down syndrome critical region gene 1	DSCR1	Up	3E−06	No
	X68487	Hs.45743	Adenosine A2b receptor	ADORA2B	Down	1E−06	No
	J03161	Hs.155321	Serum response factor (c-fos serum response transcription factor)	SRF	Down	0.001	No
	M62402	Hs.274313	Insulin-like growth factor binding protein 6	IGFBP6	Down	2E−05	Yes
	M62403	Hs.1516	Insulin-like growth factor-binding protein 4	IGFBP4	Up	0.0004	Yes
	L20861	Hs.120	Wingless-type MMTV integration site family, member 5A	WNT5A	Up	3E−05	Yes
	M34539	Hs.752	FK506-binding protein 1A (12kD)	FKBP1A/FKPB12	Up	7E−05	No

EOC=epithelial ovarian cancer.

a Mann–Whitney test.

**Table 3:** Description of 18 genes forming the ovarian tumour classifier

**Probe set HUFL**	**Unigene**	**Name**	**Symbol**	**Regulation in ovarian tumours**	**Ratio**	**P^a^**	**Classifier of malignancy**
X94232_at	Hs.78335	Microtubule-associated protein 2, RP family	RP1/MAPRE2	Down	0.6	2E−04	Invasive
X83416_s_at		PrP gene, exon 2	PRP	Up	2.1	1E−04	Invasive
X68487_at	Hs.45743	A2b adenosine receptor.	ADORA2B	Down	0.46	2E−05	Invasive
U40434_at	Hs.155981	Mesothelin or CAK1 antigen precursor mRNA	MSLN	Up	2.5	2E−04	Invasive
U16799_s_at	Hs.78629	Na,K-ATPase beta-1 subunit mRNA	ATPase*β*1	Up	4.5	2E−05	Invasive
U15131_at	Hs.79265	p126 (ST5) mRNA, complete cds.	ST-5/HTS1	Down	0.6	2E−04	Invasive
U01062_at	Hs.77515	type 3 inositol 1,4,5-trisphosphate receptor (ITPR3)	ITPR3	Up	2.7	2E−04	Invasive
M63603_at	Hs.85050	Phospholamban mRNA, complete cds.	PLN	Down	0.3	4E−05	Invasive
M16937_at	Hs.819	Homeobox c1 protein; homeobox B7	HoxB7	Up	2.5	1E−05	Invasive
HG1612-HT1612_at			MARCKS	Down	0.4	5E−05	Invasive
D28137_at	Hs.118110	BST-2	BST-2	Down	0.5	2E−04	Invasive
U79275_at	Hs.27414	Human clone 23947 mRNA	HSU79275	Up	2	0.149	LMP
U70671_at	Hs.43509	A2RP; Human ataxin-2 related protein mRNA, partial cds.	A2RP	Down	0.5	0.149	LMP
U60269_cds3_at			U606269	Up	1.5	0.149	LMP
U46006_s_at	Hs.10526	Smooth muscle lim protein	SmLIM	Down	0.6	0.149	LMP
U07664_at		HB9 homeobox gene, exons 2 and 3 and complete cds.	HoxB9	Up	2.5	0.114	LMP
M18700_s_at		Elastase III A gene, exon 8	ELIII	Up	1.9	0.093	LMP
HG830-HT830_at			HG830-Ht830	Up	1.7	0.093	LMP

Gene classifier list of 18 genes discriminating low malignant potential (LMP) tumours and invasive tumours from normal ovarian surface epithelial samples.

a Mann–Whitney test.

**Table 4:** Molecular classification of ovarian tumours used in different sets of samples

**Sample set**	**Gene classifiers**	**Correctly classified (*n*/%)**	**Errors**	**Nonclassified**	**Ratio (error/correct)**
Training set (*n*=65)	4888 genes	33/51	8	24	0.24
	130 genes	52/80	8	5	0.15
	18 genes	60/92	3	2	0.05
	130 genes	132/96	4	1	0.06
					
Test set tissue (*n*=137)	18 genes	132/96	2	3	0.03
Test set (*n*=23)	130 genes	18/78	2	3	0.11
	18 genes	20/87	1	2	0.05

*k*-neighbour class prediction of different sample sets. Cutoff 0.2.

**Table 5:** Immunohistochemical staining of an ovarian tissue array with anti-mesothelin and anti-BMP-2 antibodies

		***P* a**	**0 (*n*)**	**1+ (*n*)**	**2+ (*n*)**	**3+ (*n*)**	**4+(*n*)**
Mesothelin	Normal (*n*=20)		**7**	**6**	6	1	0
	(*n*=4)	0.01	0	0	**1**	**1**	**2**
	Grade 1 (*n*=18)	0.25	8	2	**6**	**3**	**0**
	Grade 2 (*n*=17)	0.03	7		**2**	**5**	**3**
	Grade 3 (*n*=24)	0.01	7	3	**2**	**10**	**2**
	Clear cells (*n*=17)	0.41	10	0	**3**	**4**	**0**
	Endometrioid (*n*=27)	0.25	13	3	**4**	**6**	**1**
	Serous (*n*=21)	<0.01	1	2	**3**	**9**	**6**
	Mixed (*n*=5)	0.14	1	1	**1**	**1**	**1**
	Total tumours (*n*=70)	0.07	25	6	**11**	**20**	**8**
							
BMP-2	Normal (*n*=20)		**8**	**0**	**7**	5	0
	Grade LMP serous (*n*=4)	0.25	1	1	0	**0**	**2**
	Grade 1 (*n*=18)	0.20	8	0	1	**4**	**5**
	Grade 2 (*n*=17)	<0.01	1	1	2	**3**	**10**
	Grade 3 (*n*=24)	<0.01	3	1	4	**4**	**12**
	Clear cells (*n*=17)	<0.01	1	0	1	**0**	**16**
	Endometrioid (*n*=27)	0.04	8	1	3	**5**	**10**
	Serous (*n*=21)	0.06	5	2	3	**5**	**6**
	Mixed (*n*=5)	0.25	2	0	0	**1**	**2**
	Total tumours (*n*=70)	0.05	16	3	7	**11**	**34**

Bold values highlight the positive cancerous samples and the negative normal epithelia samples.

LMP=low malignant potential.

a *t*-Test.
